# Ecotoxicity and bioremediation potential assessment of soil from oil refinery station area

**DOI:** 10.1007/s40201-021-00780-0

**Published:** 2022-01-22

**Authors:** Iwona Zawierucha, Grzegorz Malina, Barbara Herman, Piotr Rychter, Robert Biczak, Barbara Pawlowska, Katarzyna Bandurska, Renata Barczynska

**Affiliations:** 1 Jan Dlugosz University in Czestochowa, Faculty of Science & Technology, Armii Krajowej 13/15, 42-200, Czestochowa, Poland; 2 AGH University of Science and Technology, Department of Hydrogeology and Engineering Geology, Mickiewicza 30, 30-059 Cracow, Poland

**Keywords:** Bioremediation, Ecotoxicity, Oil hydrocarbons, Phytotoxicity, Respirometric test

## Abstract

**Purpose:**

The aim of the present study was to evaluate the toxicity and biodegradation potential of oil hydrocarbons contaminated soil samples obtained from different depths at an oil refinery station area. An approach involving chemical, microbiological, respirometry and ecotoxicity assessment of soil polluted by oil hydrocarbons was adopted, in order to determine the biodegradability of pollutants and ecotoxicological effects of natural attenuation strategy.

**Methods:**

The ecotoxicity of soil samples was evaluated using an ostracod test kit and a seed germination test. The results of the phytotoxicity assay were expressed as a percentage of seedling emergence and as the relative yield of fresh and dry biomass compared to control plants. The intrinsic biodegradation potential of the contaminated soil was examined using a Micro-Oxymax respirometer. Intrinsic biodegradation rates were estimated from the slopes of linear regressions curves plotted for cumulative O_2_ uptake. The obtained values were then entered in the mass balance equation for the stoichiometric reaction of hydrocarbon decomposition and converted per kg of soil per day.

**Results:**

Although the tested contaminants were biodegradable in the respirometric assay, they were slightly to moderately toxic to plants and extremely toxic to ostracods. The noxious effects raised with the increased concentration of contaminants. The monocotyledonous oat was more tolerant to higher concentrations of oil hydrocarbons than the other test plants, indicating its greater suitability for soil reclamation purposes.

**Conclusion:**

By assessing phytotoxicity and effect on ostracod mortality and progress of soil self-decontamination process, proper approach of reclamation of demoted area can be provided.

## Introduction

The quality of life on the Earth is inextricably linked to the overall status of the environment. The development of human civilization has led to growing disruptions in the natural equilibria and the emergence of diverse types of pollution [1–3].

Environmental contamination with oil (i.e. petroleum) hydrocarbons is one of the most serious ecological problems worldwide [4–6]. The increasing industrialization of the world’s economy has led to a significant increase in consumption of petrochemical compounds [2, 3]. In order to meet the growing global demand for petroleum, the world’s output of crude oil is expected to reach 95 million barrels daily [6]. It is estimated that 2.5 million tons of petroleum products, including heavy hydrocarbon fractions, are released into the environment annually. Water and soil contamination with oil hydrocarbons due to prospecting, production, handling, transport, storage, and accidental releases, represents a significant threat to humans and the environment [5, 7].

The use of crude oil as the main source of energy in the process of civilizational development has led to considerable environmental pollution, despite some undeniable economic benefits. Oil hydrocarbons penetrating into soils are a major threat to groundwater [4, 8, 9]. Hydrocarbon contamination can appear locally and periodically as a consequence of accidental leaks from damaged wells, pipelines, tanker ships/trucks/cars, as well as petroleum product storage and distribution facilities. In some areas oil hydrocarbons may also occur permanently, for instance as a consequence of the operation of oil refineries and the industry [10].

Oil hydrocarbons can be classified into four categories: aliphatics, aromatics, resins (carbazoles, sulfoxides, pyridines, quinolines, and amides), and asphaltenes (phenols, ketones, esters, porphyrins, and fatty acids) [11, 12]. The first ones are usually degraded by microorganisms, but large branched aliphatic chains and aromatic hydrocarbons usually persist in the environment. Oil hydrocarbons of particular importance include: benzene, toluene, ethylbenzene, and xylene, collectively known as BTEX and polycyclic aromatic hydrocarbons (PAHs) [6]. PAHs consist of two or more fused aromatic rings and include naphthalene, acenaphthene, fluorene, phenanthrene, and pyrene. PAHs are carcinogenic, cytotoxic, genotoxic, and environmentally noxious [13]. In a real event of oil contamination, PAHs are usually present as a mixture of several aromatic compounds, where each one of them can influence others affecting their bioavailability and increasing the difficulty for biological degradation [12].

The degree of soil contamination by oil hydrocarbons and the course of remediation of contaminated soils are usually monitored by instrumental analysis methods, such as gas chromatography and infrared spectrophotometry [14, 15]. However, the measurement of contaminant levels in a given environment does not always fully reflect the actual ecotoxicological hazards, and in recent years biological tests evaluating the ecotoxicity of contaminated soils have attracted widespread attention. Biological studies have clearly demonstrated that chemical analysis alone is not sufficient for determining the potential ecological impacts of contaminated soils [5, 16–18]. Indeed, ecotoxicological studies constitute a valuable complement to chemical tests in assessing the risks arising from the presence of hazardous substances in soil [16, 19, 20].

Biological tests have proven to be particularly useful in predicting the ecological impact of complex mixtures of compounds, such as oil hydrocarbons [5, 16, 21]. They involve phytotoxicity tests to higher plants [22], especially when the contaminants present in soil are known (or suspected) to be phytotoxic [16]. Oil hydrocarbons may cause structural deformations in cells and tissues, inhibit growth, or even lead to plant death [23]. Long-term studies enable the assessment of acute and chronic toxicity by measuring plant biomass and elongation of roots over two to eight weeks. Short-term plant growth studies primarily evaluate acute toxic effects. In germination (emergence) studies, seeds are planted in a small amount of contaminated soil, and seedlings are counted after a period of incubation and compared with seedling counts for uncontaminated soil [16]. Previous studies have shown that soil contamination with oil hydrocarbons reduces emergence and limits the growth of plants [5, 24]. However, different plants react differently to soil contaminated with petroleum products, whose effects on growth depend on the susceptibility of plant species and the degree of soil contamination [2]. The need to analyze numerous environmental samples in a relatively short time has emphasized the importance of the so-called microbiotests – rapid miniaturized toxicity tests [25]. Microbiotests involving ostracods (crustaceans) are considered cost-effective and user-friendly tools for routine monitoring of the toxicity of contaminated soil samples [26], with the commercially available kits being sold under the name of Ostracodtoxkit F™ [25].

One of the most beneficial methods of counteracting the effects of soil contamination is biological remediation [23, 27], with its three major types: natural bioremediation, biostimulation, and bioaugmentation. The first method relies on natural (intrinsic) biodegradation processes carried out by native (autochthonous) microorganisms, the second involves stimulating the growth and activity of the native population of microorganisms by providing them with additional sources of nutrients, oxygen and/or other electron acceptors to accelerate contaminant decomposition, while the third consists of introducing indigenous/extraneous/genetically modified microorganisms into the environment (soil-water system) [10, 28]. Bioremediation, which uses the existing community of microorganisms is often the most cost-effective clean-up method available. Indeed, in most cases native microbial activity may be sufficient to adequately clean up contaminated soil [27]. Soil microorganisms are capable to transform oil hydrocarbons to less- (biotransformation), non- (complete biodegradation) toxic compounds, or eventually mineralize them into simple inorganic substances such as carbon dioxide and water [27, 29]. Intrinsic biodegradation plays an important role in soil self-decontamination often referred to as natural attenuation (NA). The participation of numerous group of microorganisms from highly aerobic to completely anaerobic interacting with each other is necessary to achieve complete biodegradation of oil hydrocarbons [14, 30]. Many soil microorganisms can degrade petroleum hydrocarbons under aerobic or anaerobic conditions [14]. Microbial metabolic activity may remove certain contaminants or convert them into less aggressive substances, thereby reducing environmental risks and enabling normal enzymatic biodegradation processes responsible for environmental self-purification [1, 31].

Biodegradation of most oil hydrocarbons occurs faster and is more complete under aerobic conditions [32]. It largely depends also on the specific soil conditions and native microflora. Factors affecting biodegradation include: the concentration and chemical structure of hydrocarbons, their toxicity to the autochthonous microflora, the microbiological capacity of the soil (the qualitative and quantitative composition of microorganisms and their enzymatic activity), the physicochemical parameters of the environment (e.g., pH, temperature, redox potential, moisture content, organic matter content, and concentrations of nutrients such as nitrogen and phosphorus), as well as availability of contaminants to the microorganisms [14, 33–35].

A major role in bioremediation is played by microorganisms capable of utilizing hydrocarbons as a source of carbon and energy. The rate and effectiveness of soil bioremediation depend on the quantity and degradation activity of soil microorganisms [34], and so the bioremediation potential can be inferred from the determination of those parameters. The microbial activity of soils can be assessed by a number of methods, such as measuring soil respiration [36] or the activity of enzymes, e.g., dehydrogenase [15]. Dehydrogenase activity is an indicator of the respiratory intensity of soil microorganisms, and is thus a good proxy for soil biological activity [37].

A common method for evaluating soil microbial activity useful in assessing the decomposition of organic compounds in soil is respirometry [36, 38]. It is used, e.g., in studies on aerobic biodegradation of oil hydrocarbons in soil, with degradation rates being inferred from the curves describing O_2_ consumption and CO_2_ production [10, 29, 36]. Respirometric methods play a crucial role in cutting-edge biodegradability testing. A new generation of accurate, highly automated respirometers (e.g., Sapromat and Oxymax) has been developed [39] and successfully deployed for monitoring slow processes, such as oil hydrocarbons biodegradation rates in environmental samples [7, 10, 40, 41]. Respirometric tests have also been proposed for assessing the toxicity of soil contaminants [42–45]. Other studies have exploited in-situ respirometric techniques to measure pollution-induced microbial community tolerance in long-term contaminated soils [46–48].

Ecotoxicological tests have successfully been used as a complementary tool to monitor bioremediation efficiency in soil, which is important to assess ecological risks at polluted site [49]. However, very few studies combine these toxicological tests with a detailed study of biodegradation potential in oi hydrocarbons polluted soils. To ensure proper risk assessment of contaminated sites and the monitoring of soil self-decontamination process, toxicity assays, chemical analyses, microbial and respirometry studies of polluted area should be combined.

The aim of the present study was to evaluate the toxicity and biodegradation potential of oil hydrocarbons contaminated soil samples obtained from different depths at an oil refinery station area. An approach involving chemical, microbiological, respirometry and ecotoxicity assessment of soil polluted by oil hydrocarbons was adopted, in order to determine the biodegradability of pollutants and ecotoxicological effects of natural attenuation strategy.

## Materials and methods

### Soil samples

Samples of soil contaminated with oil hydrocarbons originated from the oil refinery station in Czechowice-Dziedzice, Poland. The samples were collected from depths of 0–0.5 m (R1) and 1.0–1.5 m (R2). An uncontaminated (reference) soil sample was also collected (R0).

#### Hydrocarbons content in soil

The hydrocarbons content in soil was determined using the headspace method and capillary gas chromatography with mass detection (GC-MS). A Shimadzu GC-14A gas chromatograph was coupled to a Shimadzu QP 5000 mass spectrometer. Analyses were made according to the standard ISO 16703:2009 [50].

#### Physicochemical, biochemical and microbial investigations of soil samples

The pH of soil suspensions was determined according to the standard ISO 10390:1997 [51], soil moisture – ISO 11465:1999 [52], soil organic matter (SOM) – ISO 10694:2002 [53], nitrogen content – ISO 11261:2002 [54], and phosphorus content – ISO 11263:2002 [55].

Bacterial populations were measured in terms of colony-forming units (CFU) per gram by means of the standard agar plate technique. A series of dilutions was made form soil suspension with sterile distilled water, with the same volume of each dilution being plated on agar medium (20.0 g of agar and 15.0 g of nutrient broth in 1.0 L of distilled water). The bacteria were incubated at 28 °C for 48 h.

The microbial activity was measured using triphenyltetrazolium chloride (TTC) as an artificial electron acceptor to estimate dehydrogenase activity, as the TTC reduction to triphenyl formazan (TPF) causes a color change that can be quantified spectrophotometrically. Soil samples of 1 g were incubated with 3% TTC at 37 °C for 24 h. Then, the samples were extracted with methanol and the soil suspension was filtered. Finally, the optical density of the clear supernatant was measured against a blank sample at 546 nm using a UV-VIS spectrophotometer (Varian), with the results presented as TPF mg per gram dry weight of soil [56].

### The ecotoxicity of studied soil samples

#### Phytotoxicity - the plant growth inhibition test

The phytotoxic potential of oil hydrocarbons was determined in a pot experiment carried out in a vegetation hall [57], based on the OECD Guidelines 208/2006 [58] and the standard ISO 11269-2:2012 [59]. The oat (*Avena sativa*), garden cress (*Lepidium sativum* L.), and common radish (*Raphanus sativus* L. subvar. Radicula Pers.) were used in the experiment. Plastic plant pots with a diameter of 90 mm were filled with contaminated soils (samples R1 and R2) and a reference soil (sample R0). Subsequently, 20 identical seeds of oat, 20 seeds of radish, and 50 seeds of garden cress were sown in each pot. The plants were grown for 2 weeks under controlled conditions with soil moisture, light intensity, and temperature being maintained at a stable level.

To evaluate the phytotoxicity of oil hydrocarbons, the percentage emergence and weight (dry and fresh) of control plant seedlings (R0) were determined and compared with the corresponding parameters measured for the plants grown in contaminated soil (R1, R2). The visual assessment of any damage to the test plants was also carried out and documented by digital photographs.

All experimental results were statistically analyzed using STATISTICA. Data from four measurements (*n* = 3) were analyzed using analysis of variance ANOVA followed by Tukey’s post-hoc test. Least significant differences (LSD) were determined at *p* < 0.05.

#### The Ostracod test kit

The toxicity of soil samples was determined in a short-term contact test by means of an Ostracodtoxkit F (MicroBioTests Inc., Nazareth, Belgium), which is a solid-phase microbiotest for freshwater sediments and soils employing newly hatched *Heterocypris incongruens* ostracods (freshwater crustaceans).

Each well of the test plate was filled in the following order: 2 mL of distilled water, 2 mL of algal suspension, and 700 mg of dried and sieved soil. The test plate was sealed with Parafilm, covered by a lid, and incubated at 25 °C in the dark. After 6 days, the mortality and growth inhibition of the test organisms were determined. The Ostracod length was measured using a micrometric strip placed under the glass microscope plate [26, 60], according to the Standard operational procedure: Ostracodtoxkit F [61].

### The respirometric test

**Fig. 1:**
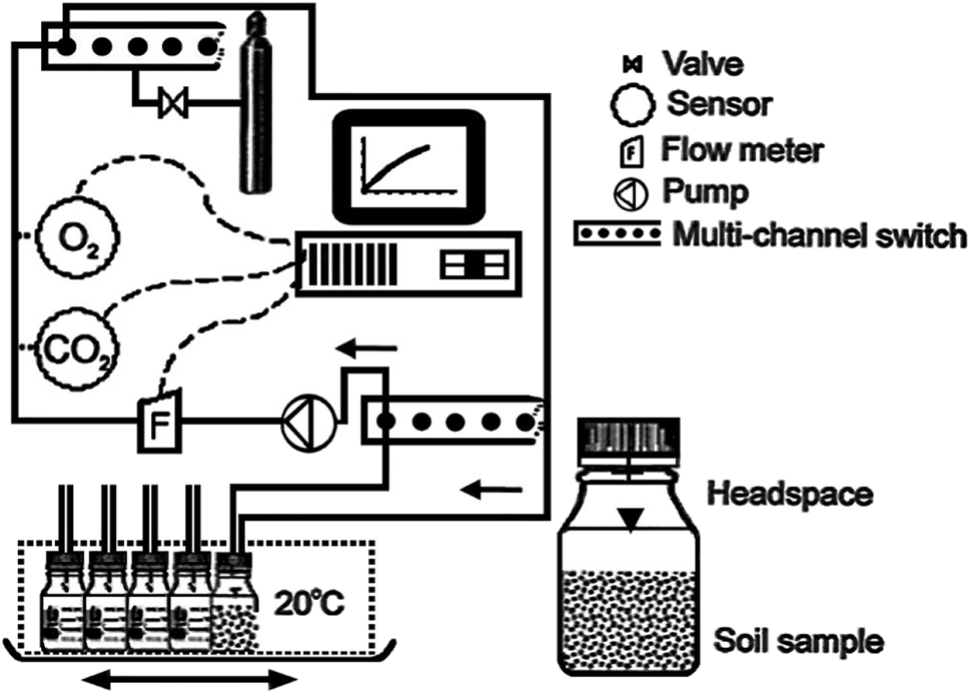
The Micro-Oxymax respirometer setup

Biodegradation of oil hydrocarbons under aerobic conditions was analyzed using a 10-chamber Micro-Oxymax respirometer (Columbus Instruments, OH, USA) capable of measuring on-line the O_2_ consumed and CO_2_ produced during oil hydrocarbons breakdown even at extreme consumption/production rates (Fig. 1). The respirometer was coupled to the computer for collecting and recording data in a fully automated approach. The Micro-Oxymax respirometer is a closed-circuit apparatus: the patented principle of measurement involves air sampling from the headspace of the sample chamber, circulating it through the gas analyzer and returning back to the sample chamber, without any contact with the sample [36].

Soil samples of 30 g were placed in the test chambers of the respirometer and a constant temperature of approx. 20 °C was maintained. The O_2_ consumption and CO_2_ production (in μL min^−1^) rates were simultaneously monitored online every hour during a 24 h period. Cumulative curves were plotted as the O_2_/CO_2_ content in the headspace versus time. The O_2_ uptake and CO_2_ production rates were represented by linear regression curves plotted for cumulative values. The Uncontaminated soil sample (R0) was used to determine basal respiration resulting from organic matter decomposition in soil.

## Results and discussion

### The oil hydrocarbon content in soil

According to the Polish Guidelines concerning earth quality (Regulation of the Environment Minister of 1 September 2016) [62], the maximum allowable concentrations of hydrocarbons in soil at industrial sites are: 750 mg/kg of dry matter for total gasoline hydrocarbons (C6-C12), and 3000 mg/kg of dry matter for total oil hydrocarbons (C12-C35).

The contents of oil hydrocarbons in soil samples are listed in Table 1. Sample R1 was severely contaminated with as the limits of total gasoline and oil hydrocarbons were exceeded. The total oil hydrocarbons content in soil sample R1 was more than three times higher than that in sample R2. Considerable contamination of refinery areas with oil hydrocarbons has been found in other studies [17, 30, 36], which also reported a decrease in hydrocarbon contents with increasing depth attributable to soil self-purification [10, 41].

**Table 1:** Contents of oil hydrocarbons in studied soil samples

The content of hydrocarbons	R1 (mg kg^−1^_d.m._)	R2 (mg kg^−1^_d.m.)_
Total gasoline hydrocarbons (C6-C12)	1200	57
Total oil hydrocarbons (C12-C35)	9601	3014

### Ecotoxicity of soil contaminated with oil hydrocarbons

The phytotoxicity was determined by comparing the seedling emergence and fresh weight yield of plants growing on oil hydrocarbons contaminated soil with the corresponding parameters for plants growing on uncontaminated soil. The results of phytotoxic effects for oat, radish, and garden cress are presented in Table 2.

**Table 2:** Changes of selected parameters in the phytotoxicity test of oil hydrocarbons contaminated soil samples (R1, R2) as compared to control (R0)

Soil sample	Number of plant seeds	Number of plant seedlings	Plant seedlings compared to control	Crop fresh weight [g/pot]	Crop fresh weight compared to control	Weight of single plant [g]	Weight of single plant compared to control	Dry weight [g/g _f.m._]	Dry weight compared to control
Oat (*Avena sativa)*									
R0	20	20	100%	2.294	100%	0.117	100%	0.087	100%
R1	20	16	80%	0.254	11%	0.016	14%	0.173	199%
R2	20	20	100%	1.605	70%	0.080	69%	0.112	129%
		LSD_0.05_=2		LSD_0.05_=0.252		LSD_0.05_=0.014		LSD_0.05_=0.07	
Radish (*Raphanus sativus* L. subvar. *Radicula* Pers)									
R0	20	18	100%	2.343	100%	0.128	100%	0.094	100%
R1	20	0	0%	0	0%	0	0%	0	0%
R2	20	19	106%	2.347	100%	0.123	97%	0.093	99%
		LSD_0.05_=1		LSD_0.05_=0.408		LSD_0.05_=0.017		LSD_0.05_=0.09	
Garden cress (*Lepidium sativum* L.)									
R0	50	44	100%	0.771	100%	0.017	100%	0.089	100%
R1	50	0	0%	0	0%	0	0%	0	0%
R2	50	46	105%	0.894	116%	0.019	111%	0.105	118%
		LSD_0.05_=2		LSD_0.05_=0.016		LSD_0.05_=0.020		LSD_0.05_=0.011	

Least significant differences (LSD) were determined at *p* < 0.05

**Fig. 2:**
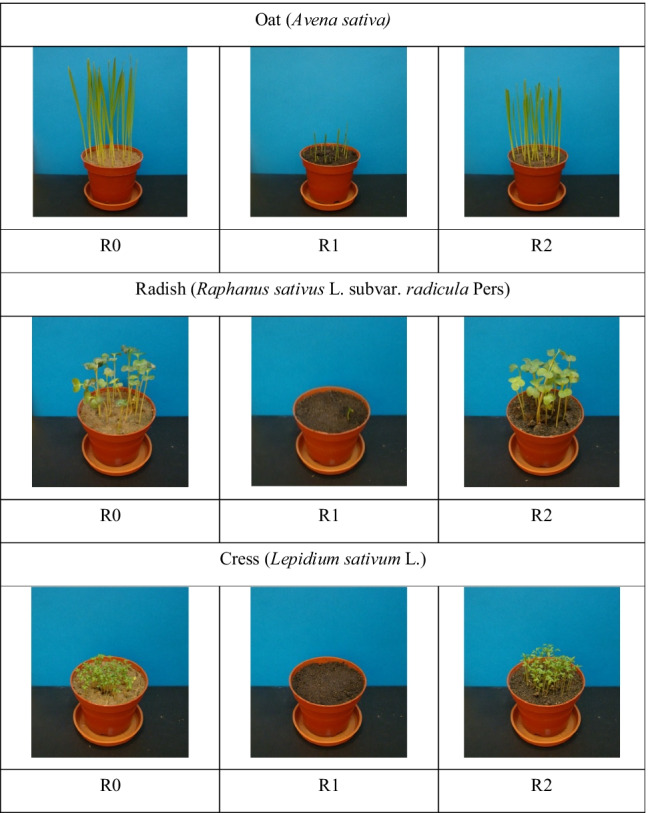
Digital photographs of oat, radish and cress seedlings in the plant growth test for contaminated soil samples (R1, R2) as compared to control (R0)

The obtained results clearly show the effects of contamination severity on soil toxicity to plants. However, while the R1 sample was much more toxic than R2 (Fig. 2), the phytotoxicity responses of the tested plant species differed. In the case of R1 sample a complete emergence failure was observed both for radish and cress. The oat emergence was of 80%, but its fresh weight yield was only of 11% as compared to control. In the case of sample R2, no plant species exhibited lower emergence, but a 30% decrease in fresh weight yield was noted vs. control. Literature reports indicate that plants vary in their sensitivity depending on the type of contamination, and so it is necessary to identify most useful species for studying the phytotoxicity of oil hydrocarbon contaminated soils [16, 63]. The present findings show that while the oat is not appropriate for phytotoxicity evaluation of oil hydrocarbons in soils in emergence tests, it is highly sensitive in fresh biomass studies, and exhibits an increase in dry weight. Similar results were obtained by Hentati et al. [5], who observed some beneficial effects of plant exposure to oil hydrocarbons in moderately contaminated soils on the total dry weight yield. However, the emergence test showed a strong response of garden cress and radish to higher concentrations of petroleum substances. The high sensitivity of L. sativum to PAHs contamination of soil has been reported previously, indicating the possibility of using this plant in assessing the toxicity of soil contaminated with these hydrocarbons [60]. A comparison of results for samples R1 and R2 proves that oil hydrocarbons affect the plant growth in a concentration-dependent manner [2] as the phytotoxicity of contaminated decreases with the degree of soil self-purification [23].

The responses of two plant species, alfalfa (*Medicago sativa*) and bristle grass (*Setaria uiridis* Beauv), to soil contaminated with oil hydrocarbons at five levels between 0 and 2.0% (w/w) were investigated by Xie et al. [64]. Their study showed that the total, aboveground and underground plant biomasses of both species were significantly reduced by oil hydrocarbons contamination, with the inhibition enhanced with increased petroleum levels. Brzeszcz et al. [65] reported that the range of phytotoxicity responses of soil historically contaminated with aliphatic and polycyclic aromatic hydrocarbons varies for different plant species. The trend—the lower content of residual pollution, the lower the inhibition of both germination and root elongation—was observed by them for *Sorghum saccharatum*, *Lepidium sativum*, and *Sinapis alba*. Among these species, *Lepidium sativum* was the most sensitive to oil hydrocarbon contamination likewise in our study.

Bes et al. [66] noted that soil contamination with oil hydrocarbons induces various changes in the morphological and physiological properties of European beeches and Scots pine. Scots pines were taller and had a larger stem diameter than European beeches. Moreover, the biomass yield of Scots pines was not significantly correlated with increasing concentrations of oil hydrocarbons, but it was more than 700% higher than in European beeches.

**Fig. 3:**
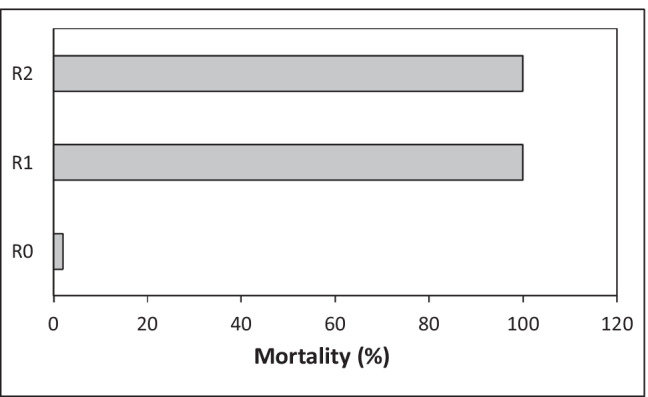
The mortality of ostracods *Heterocypris incongruens* in contaminated soil samples (R1, R2) compared to control (R0)

Toxicity assessment of contaminated soil requires knowledge of the sensitivity of the tested organisms [16]. The literature data indicate that plants have a significantly higher tolerance to oil hydrocarbons than animals [16, 23]. Also in this study, the chronic toxicity test conducted using an Ostracodtoxkit F showed 100% mortality of *Heterocypris incongruens* (Fig. 3) for both: R1 and R2 soil samples. In the reference sample, the average ostracod mortality was of 2% (± 4) with an average increase in body length of 530 μm (± 78) which proves that the test was performed correctly (Standard operational procedure: Ostracodtoxkit). The high sensitivity of *H. incongruens* ostracods to oil hydrocarbons contaminated soils has also been reported previously for PAH, with the mortality reaching 100% [26, 67]. In turn direct contact with soil from a historically heavily hydrocarbon-polluted area led to only a 54.6% ostracod mortality [65].

### The soil potential for oil hydrocarbon biodegradation

The measured physicochemical parameters of the studied soil samples (Table 3) are considered optimal for aerobic biodegradation of oil hydrocarbons [68].

**Table 3:** Soil characteristics, dehydrogenase activity and numbers of bacteria

Soil sample	pH	Moisture (%)	SOM (%)	N (mg kg^−1^_d.m._)	P (mg kg^−1^_d.m._)	Number of bacteria (CFU g^−1^)	Dehydrogenase activity (mg TPF g^−1^_d.m._)
R1	7.2	30.05	28.57	2100	146	6×10^7^	8.43
R2	7.1	25.60	12.94	1435	163	5×10^7^	8.40
R0	6.8	20.38	3.98	917	130	2×10^5^	3.54

The rate and effectiveness of bioremediation of oil hydrocarbons contaminated soils depend on the quantity and activity of soil microorganisms [34]. The minimum microbial count for effective bioremediation of such soil is 10^5^ cells/g of soil. Bacterial counts in contaminated soil samples were 250–300-fold greater than in the control sample (R0). A significant increase in the microbial population was caused by the multiplication of native microflora, which used oil hydrocarbons as a carbon source [14]. The initial number of microorganisms (10^7^ CFU g^−1^) in contaminated soils was sufficient for intrinsic biodegradation [10, 41, 69].

Soil contaminated samples (R1, R2) revealed high activity of dehydrogenases at approx. 8 mg TPF g^−1^_d.m._ (Table 3), which was more than double the dehydrogenase activity of the uncontaminated sample (R0). This is consistent with the results reported by Plaza et al. [17, 20], where high microflora respiratory activity (5.4–56.2 mg TPF × g^−1^_d.m._) was observed in soil heavily contaminated with oil hydrocarbons.

**Fig. 4:**
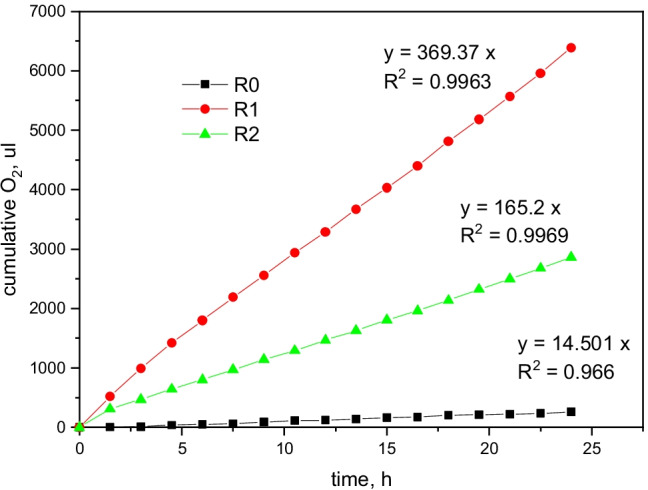
Cumulative curves of the O_2_ uptake during biodegradation of oil hydrocarbons in contaminated soil samples (R1, R2) compared to control (R0)

Biodegradation of oil hydrocarbons in tested soil was evaluated based on the O_2_ consumption, which is considered more reliable than the CO_2_ evolution because the latter could also result from the dissolution of soil carbonates. Cumulative O_2_ consumption curves were plotted as O_2_ content in the headspace versus time (Fig. 4), and O_2_ uptake rates were estimated from the slopes of their linear regressions. The coefficients (a) of linear regression equations (y=a^.^x) represented the mean rates of O_2_ consumption during oil hydrocarbons biodegradation. The applied linear regression model exhibited an excellent fit to experimental data with regression coefficients of R^2^ ≥ 0.99.

Biodegradation rates represented by the O_2_ uptake were the lowest in the uncontaminated soil sample (R0). The higher mean rates found for contaminated soil (6.16 μL min^−1^ and 2.75 μL min^−1^ for R1 and R2, respectively) indicate aerobic biodegradation of oil hydrocarbons The mean O_2_ consumption rate was higher in the shallower soil layer as compared to the deeper layer, which implies higher hydrocarbon concentrations and microbial activity in the former, resulting in a more intensive biodegradation.

Biodegradation rates were quantified based on the mean O_2_ consumption rates, assuming complete mineralization (the only products of hydrocarbon biodegradation: biomass, carbon dioxide, and water).

The mass balance was calculated from an equation describing the stoichiometric reaction of hydrocarbon decomposition:

1 $${\mathrm{C}}_{\mathrm{n}}{\mathrm{H}}_{\mathrm{m}}+a\ {\mathrm{O}}_2=Y\ {\mathrm{C}\mathrm{H}}_2\mathrm{O}+b\ {\mathrm{C}\mathrm{O}}_2+c\ {\mathrm{H}}_2\mathrm{O}$$

where: *m* – number of hydrogen atoms, *n* – number of carbon atoms, *a*, *b*, and *c* – stoichiometric coefficients of reaction, and *Y* – microbial yield.

Assuming a hydrocarbon formula of CH_1.5_ [31, 36] and a microbial yield of *Y*=0.5, the biodegradation rates (*X*) were calculated according to the following equation [36]:

2 $$X=2.144\frac{4\left(12+\frac{m}{n}\ \right)}{4\left(1-Y\right)+\frac{m}{n}}\ {k}_{o_2}$$

where: *X* – intrinsic biodegradation rate calculated on the basis of the O_2_ consumption rate [mg of hydrocarbons kg of soil^−1^ day^−1^], *k*o_2_– the O_2_ consumption rate [μL min^−1^], 2.144 – the conversion coefficient for the O_2_ consumption rate (converting Micro-Oxymax respirometer readings to [mmol of O_2_ kg of soil^−1^ day^−1^]).

The “net” values of O_2_ consumption (excluding basal respiration due to organic matter decomposition) obtained for the contaminated soil samples were used in formula (2) to calculate the actual hydrocarbon biodegradation rates. The results revealed that the biodegradation potential of R1 was much higher than R2: the mean hydrocarbon biodegradation rate for the former was of 196 mg HC kg ^−1^ day^−1^ as compared to 83 mg HC kg dw^−1^ day^−1^ for the latter. The critical factors enabling the high biodegradation rate were the high oil hydrocarbon concentration and substrate bioavailability. Indeed, Bartha [70] noted that the composition and inherent biodegradability of oil hydrocarbons is the first and foremost consideration when the suitability of a biodegradative cleanup approach is to be evaluated. Biodegradation of oil hydrocarbons is usually rapid and extensive as long as favorable conditions prevail.

## Conclusions

In the present study, plant germination, ostracod and respirometry tests were used to determine the ecotoxicity and bioremediation potential of soil contaminated with oil hydrocarbons. The pot experiments indicate that oil hydrocarbons in soil may impair the growth and development of higher land plants. The toxic effects were primarily dependent on the hydrocarbons concentration and used plant species. At the higher hydrocarbons concentration radish and cress failed to germinate. On the other hand, the ostracod mortality was of 100% for both contaminated soil samples. This indicates that even at concentrations that are nontoxic to plants hydrocarbons may critically disrupt the cellular metabolism of other living organisms. Respirometry showed a significant increase of oxygen consumption in contaminated soil samples as compared to control, which proves that the investigated soil has the potential for oil hydrocarbons biodegradation. Thus, the O_2_ consumption rates determined by means of a Micro-Oxymax respirometer can be used to accurately assess microbial activity and the degree of hydrocarbon biodegradation during bioremediation. Although the tested contaminants were biodegradable in the respirometric assay, they were slightly to moderately toxic to plants and extremely toxic to ostracods. Microbiotests are thus a useful complement to chemical analyses in terms of evaluating soil contamination and the course of bioremediation. Toxicity tests may have a good application potential as environmental monitoring tools to assess the effectiveness of soil remediation technologies. By assessing phytotoxicity and effect on ostracod mortality and progress of soil self-decontamination process, proper approach of reclamation of demoted area can be provided.
